# Supplementary material for: Insecticide resistance of Miami-Dade Culex quinquefasciatus populations and initial field efficacy of a new resistance-breaking adulticide formulation
Source: PLoS One. 2024 Feb 12;19(2):e0296046. doi: 10.1371/journal.pone.0296046 (PMC10861066; doi:10.1371/journal.pone.0296046)
Supplement: S1 Table — (DOCX) [file pone.0296046.s002.docx]

| **Supplemental Table 1: *Culex quinquefasciatus* collection information** | | | |  |  |
| --- | --- | --- | --- | --- | --- |
| Latitude | Longitude | Location | Neighborhood | | Collection Date |
| 25.437 | -80.483 | SW 355th St, Homestead, FL 33034 | Florida City | | 8/5/2020-9/2/2020 |
| 25.446 | -80.456 | SE 24th Ct, Homestead, FL 33035 | Homestead | | 9/29/2020-10/1/2020 |
| 25.517 | -80.493 | SW 268th St, Homestead, FL 33031 | Homestead | | 9/29/2020-10/1/2020 |
| 25.520 | -80.553 | SW 227th Ave, Homestead, FL 33031 | Homestead | | 11/18/2020 |
| 25.566 | -80.54 | SW 216th St, Miami, FL 33170 | Miami | | 11/4/2020-11/18/2020 |
| 25.588 | -80.313 | SW 193rd Lane, Miami, FL 33157 | Cutler Bay | | 8/31/2020-9/02/2020 |
| 25.610 | -80.536 | SW 212th Ave, Miami, FL 33187 | Miami | | 11/4/2020 |
| 25.636 | -80.372 | SW 110th Ave, Miami, FL 33176 | Richmond Heights | | 9/8/2020-9/10/2020 |
| 25.637 | -80.294 | SW 144th St, Palmetto Bay, FL 33158 | Coral Gables | | 8/31/2020-9/2/2020 |
| 25.639 | -80.516 | SW 136th St, Miami, FL 33196 | Miami | | 11/17/2020 |
| 25.657 | -80.285 | Moss Ranch Rd, Pinecrest, FL 33156 | Pinecrest | | 8/5/2020 |
| 25.711 | -80.34 | SW 62nd Terrace, Miami, FL 33173 | Miami | | 7/6/2022 |
| 25.754 | -80.337 | SW 87th Pl, Miami, FL 33165 | Westchester | | 9/8/2020-9/9/2020 |
| 25.790 | -80.206 | NW 6th Pl, Miami, FL 33136 | Town Park | | 7/30/2020 |
| 25.801 | -80.134 | Prairie Ave, Miami Beach, FL 33140 | Bayshore | | 7/20/2022 |
| 25.805 | -80.196 | NW 30th St, Miami, FL 33127 | Wynwood | | 8/18/2020 |
| 25.812 | -80.417 | NW 41st St, Miami, FL 33182 | West Doral | | 8/5/2020 |
| 25.814 | -80.226 | NW 42nd St, Miami, FL 33142 | Allapattah | | 9/11/2020-9/20/2020 |
| 25.827 | -80.342 | NW 58th St, Miami, FL 33178 | Doral | | 8/5/2019 |
| 25.834 | -80.129 | Alton Rd, Miami Beach, FL 33140 | La Gorce | | 10/14/2020 |
| 25.853 | -80.131 | Bay Dr, Miami Beach, FL 33141 | Normandy Island | | 8/12/2020-8/13/2020 |
| 25.862 | -80.313 | W 44th St, Hialeah, FL 33012 | Hialeah | | 9/9/2020-9/10/2020 |
| 25.893 | -80.258 | Cairo Ln, Opa-locka, FL 33054 | Opa-Locka | | 10/28/2020 |
| 25.897 | -80.126 | Park Dr, Bal Harbour, FL 33154 | Bal Harbour | | 10/5/2020 |
| 25.905 | -80.25 | Caliph St, Opa-locka, FL, 33054 | Opa-Locka | | 10/28/2020 |
| 25.911 | -80.355 | Altis Cir W, Hialeah, FL 33018 | Hialeah | | 10/28/2020 |
| 25.930 | -80.216 | NW 170th Terrace, Miami, FL 33169 | Scott Lake | | 9/29/2020 |
| 25.938 | -80.345 | NW 181st St, Hialeah, FL 33018 | Hialeah | | 10/29/2020 |
| 25.968 | -80.214 | NW 7th Ave, Miami Gardens, FL 33169 | Miami Gardens | | 9/29/2020 |
