# Supplementary material for: Insecticide resistance of Miami-Dade Culex quinquefasciatus populations and initial field efficacy of a new resistance-breaking adulticide formulation
Source: PLoS One. 2024 Feb 12;19(2):e0296046. doi: 10.1371/journal.pone.0296046 (PMC10861066; doi:10.1371/journal.pone.0296046)
Supplement: S2 Table — (DOCX) [file pone.0296046.s003.docx]

| **Table S2: Miami-Dade County *Cx. quinquefasciatus* mortality at diagnostic times and 120 minutes of exposure in CDC bottle bioassays against multiple active ingredients** | | | | | | | | |
| --- | --- | --- | --- | --- | --- | --- | --- | --- |
| Location | Mortality (%) at Diagnostic Time | | | | Mortality (%) at 120 minutes | | | |
|  | Permethrin @ 30 min | Deltamethrin @ 60 min | Malathion @ 45 min | Naled @ 45 min | Permethrin | Deltamethrin | Malathion | Naled |
| SW 355th St, Homestead | 7 | 11.3 | 47.8 | n.d. | 47 | 24.5 | 97.8 | n.d. |
| SE 24th Ct, Homestead | 12.9 | 12 | 62.7 | 76.8 | 51.4 | 17.3 | 83.6 | 97.1 |
| SW 268th St, Homestead | 2.7 | 0 | 52.4 | 89.1 | 50.7 | 2 | 59 | 98.4 |
| SW 227th Ave, Homestead | 0 | 1.8 | 30 | 0 | 39.3 | 17.9 | 64 | 54.8 |
| SW 216th St, Miami | 2.7 | 4.1 | 61.6 | 63.6 | 41.1 | 20.4 | 84.9 | 73.4 |
| SW 193rd Lane, Miami | 18.3 | 5 | 91.7 | 97.6 | 81.7 | 13 | 98.6 | 100 |
| SW 212th Ave, Miami | 19 | 11.3 | 80 | 32.7 | 84.1 | 24.2 | 92.8 | 94.3 |
| SW 110th Ave, Miami | 27.1 | 9.4 | 79.2 | 98.4 | 72.9 | 18.8 | 94.9 | 100 |
| SW 144th St, Palmetto Bay | 0 | 0 | 90.4 | 100 | 63.5 | 3.6 | 94.2 | 100 |
| SW 136th St, Miami | 39.1 | 16.5 | 40.6 | 80.4 | 89.9 | 32.9 | 81.25 | 100 |
| Moss Ranch Rd, Pinecrest | 20 | 11.4 | 89.4 | 95.7 | 71.1 | 20 | 89.4 | 100 |
| SW 62nd Terrace, Miami | 59 | 5.3 | 100 | n.d. | 96.7 | 39.5 | 100 | n.d. |
| SW 87th Pl, Miami | 20.5 | 18.8 | 95.7 | 94.8 | 67.1 | 24.4 | 94.2 | 100 |
| NW 6th Pl, Miami | 40 | 14.7 | 100 | n.d. | 80 | 35.3 | 100 | n.d. |
| Prairie Ave, Miami Beach | n.d. | n.d. | 100 | 92.9 | n.d. | n.d. | 100 | 100 |
| NW 30th St, Miami | 75.6 | 25.2 | 97.7 | n.d. | 84.9 | 77.4 | 100 | n.d. |
| NW 41st St, Miami | 19.4 | 15 | 100 | n.d. | 51.6 | 25 | 100 | n.d. |
| NW 42nd St, Miami | 57 | 28.5 | 94 | 100 | 97 | 55 | 100 | 100 |
| NW 58th St, Miami | 17.1 | 0 | 100 | n.d. | 73.2 | 5.2 | 100 | n.d. |
| Alton Rd, Miami Beach | 17 | 26.5 | 97.4 | n.d. | 86 | 36.8 | 100 | n.d. |
| Bay Dr, Miami Beach | 34.6 | 2.6 | 89.7 | n.d. | 59 | 10.4 | 100 | n.d. |
| W 44th St, Hialeah | 6.9 | 8.8 | 87.7 | 100 | 34.5 | 11.8 | 98.2 | 100 |
| Cairo Ln, Opa-locka | n.d. | 9.4 | 44.4 | n.d. | n.d. | 26.4 | 100 | n.d. |
| Park Dr, Bal Harbour | 17 | 9 | 90.6 | 96.2 | 84.2 | 23.9 | 100 | 100 |
| Caliph St, Opa-locka | 17 | 50.8 | 97.6 | 84 | 83.3 | 58.5 | 100 | 100 |
| Altis Cir W, Hialeah | 36.4 | 36.5 | 86.8 | 90.5 | 59.1 | 57.7 | 100 | 100 |
| NW 170th Terrace, Miami | 35 | 11.4 | 97.1 | 100 | 72.5 | 28.6 | 100 | 100 |
| NW 181st St, Hialeah | 31.3 | 24.5 | 98.2 | 91.5 | 68.8 | 32.6 | 100 | 100 |
| NW 7th Ave, Miami Gardens | 32.7 | 20 | 90.7 | 98.8 | 69.4 | 24.7 | 100 | 100 |
