# Supplementary material for: Insecticide resistance of Miami-Dade Culex quinquefasciatus populations and initial field efficacy of a new resistance-breaking adulticide formulation
Source: PLoS One. 2024 Feb 12;19(2):e0296046. doi: 10.1371/journal.pone.0296046 (PMC10861066; doi:10.1371/journal.pone.0296046)
Supplement: S3 Table — (DOCX) [file pone.0296046.s004.docx]

**Table S3: Genotyping and Allele Percentages for the leucine to phenylalanine 1014 mutation in Miami-Dade County *Cx. quinquefasciatus* populations**

| Location | Genotype percentage | | | Allele Percentage | |
| --- | --- | --- | --- | --- | --- |
|  | LL | LF | FF | L | F |
| SW 355th St, Homestead | n.d. | n.d. | n.d. | n.d. | n.d. |
| SE 24th Ct, Homestead | 23.8 | 52.4 | 23.8 | 50.0 | 50.0 |
| SW 268th St, Homestead | 35.9 | 30.8 | 33.3 | 51.3 | 48.7 |
| SW 227th Ave, Homestead | n.d. | n.d. | n.d. | n.d. | n.d. |
| SW 216th St, 30, Miami | n.d. | n.d. | n.d. | n.d. | n.d. |
| SW 193rd Lane, Miami | 27.1 | 58.3 | 14.6 | 56.3 | 43.8 |
| SW 212th Ave, 30, Miami | n.d. | n.d. | n.d. | n.d. | n.d. |
| SW 110th Ave, Miami | 23.4 | 61.7 | 14.9 | 54.3 | 45.7 |
| SW 144th St, Palmetto Bay | 19.1 | 66.0 | 14.9 | 52.1 | 47.9 |
| SW 136th St, 30, Miami | n.d. | n.d. | n.d. | n.d. | n.d. |
| Moss Ranch Rd, Pinecrest | 11.4 | 43.2 | 45.5 | 33.0 | 67.0 |
| SW 62nd Terrace, Miami | n.d. | n.d. | n.d. | n.d. | n.d. |
| SW 87th Pl, Miami | 40.9 | 56.8 | 2.3 | 69.3 | 30.7 |
| NW 6th Pl, Miami | 29.8 | 48.9 | 21.3 | 54.3 | 45.7 |
| Prairie Ave, Miami Beach | n.d. | n.d. | n.d. | n.d. | n.d. |
| NW 30th St, Miami | 50.0 | 50.0 | 0.0 | 75.0 | 25.0 |
| NW 41st St, Miami | 36.7 | 63.3 | 0.0 | 68.3 | 31.7 |
| NW 42nd St, Miami | 83.3 | 16.7 | 0.0 | 91.7 | 8.3 |
| NW 58th St, Miami | n.d. | n.d. | n.d. | n.d. | n.d. |
| Alton Rd, Miami Beach | 48.6 | 51.4 | 0.0 | 74.3 | 25.7 |
| Bay Dr, Miami Beach | 47.9 | 47.9 | 4.2 | 71.9 | 28.1 |
| W 44th St, Hialeah | 62.1 | 37.9 | 0.0 | 81.0 | 19.0 |
| Cairo Ln, Opa-locka | n.d. | n.d. | n.d. | n.d. | n.d. |
| Park Dr, Bal Harbour | 34.1 | 58.5 | 7.3 | 63.4 | 36.6 |
| Caliph St, Opa-locka | n.d. | n.d. | n.d. | n.d. | n.d. |
| Altis Cir W, Hialeah | n.d. | n.d. | n.d. | n.d. | n.d. |
| NW 170th Terrace, Miami | 34.9 | 51.2 | 14.0 | 60.5 | 39.5 |
| NW 181st St, Hialeah | n.d. | n.d. | n.d. | n.d. | n.d. |
| NW 7th Ave, Miami Gardens | 32.4 | 64.9 | 2.7 | 64.9 | 35.1 |
