# Supplementary material for: Insecticide resistance of Miami-Dade Culex quinquefasciatus populations and initial field efficacy of a new resistance-breaking adulticide formulation
Source: PLoS One. 2024 Feb 12;19(2):e0296046. doi: 10.1371/journal.pone.0296046 (PMC10861066; doi:10.1371/journal.pone.0296046)
Supplement: S4 Table — (DOCX) [file pone.0296046.s005.docx]

| **Table S4. Weather during ReMoa Tri ground ULV field trial.** | | | |
| --- | --- | --- | --- |
| Weather Station Height (ft) | Temperature (F) | Relative Humidity (RH%) | Wind Speed (mph) |
| 5 | 81.1 | 85.2 | 3.5 |
| 30 | 81.2 | 88.4 | 4 |
