# Supplementary material for: Insecticide resistance of Miami-Dade Culex quinquefasciatus populations and initial field efficacy of a new resistance-breaking adulticide formulation
Source: PLoS One. 2024 Feb 12;19(2):e0296046. doi: 10.1371/journal.pone.0296046 (PMC10861066; doi:10.1371/journal.pone.0296046)
Supplement: S5 Table — (DOCX) [file pone.0296046.s006.docx]

| **Table S5. Droplet parameters from ReMoa Tri ground ULV field trial** | | |
| --- | --- | --- |
| Distance from sprayer (meter) | VMD (µm) | Droplet density (per mm²) |
| 30.5 | 15.7 ± 1.5 | 23.3 ± 8.1 |
| 70 | 17.3 ± 3.8 | 30.3 ± 15.3 |
| 91.4 | 15.3 ± 3.1 | 15.7 ± 12.1 |
