# Supplementary material for: Insecticide resistance of Miami-Dade Culex quinquefasciatus populations and initial field efficacy of a new resistance-breaking adulticide formulation
Source: PLoS One. 2024 Feb 12;19(2):e0296046. doi: 10.1371/journal.pone.0296046 (PMC10861066; doi:10.1371/journal.pone.0296046)
Supplement: Attachment — Submitted filename: Response to Reviewers.docx [file pone.0296046.s008.docx]

Response to Reviewers:

Reviewer #1: The study designs, amount of work and paper writing are appropriate and effective. The result of the study is of high value to vector control workers. Paper reads nice. Overall, this is a nice piece of work, and should be published.

Reviewer #2: Thank you for addressing the reviewer comments. There are a few minor edits that I'm recommending that are indicated below.

Including Table 1 in the body of the manuscript rather supplementary information remains questionable to me. It's not a measurement, and while the information is essential for the study, it can be looked up in supplementary information for readers that want those details. Additionally, the sites are indicated on the maps of Figure 1 and 2. I think moving it to supplementary is reasonable, but I can see authors perspective that moving it results in 3 figures and 1 table in the body of the manuscript.

**Author Response:** **Considering the Reviewer comments, we have removed Table 1 and placed it in as a supplemental table. The text has been edited to accommodate this change.**

Line 147: please indicate here if CMAVE strain is susceptible or resistant, and to which insecticide class. **Author Response: The CMAVE strain is a laboratory susceptible strain and this is stated in Line 148. The tox profile of this strain has been widely examined so we have added a citation.**

206: remove additional "." **Author Response: Accepted**

249: this sentence is grammatically incorrect, and doesn't make sense as written. Please rephrase. **Author Response: Sentence revised.**

319 - 323: Data here repeats verbatim what is shown in Table 2. To avoid repetition, consider converting Table 2 to a histogram (distance on the x- axis, with bars side-by-side for 24 h and 48h time points, y-axis as % mortality). Alternatively, consider summarizing the data in the body of the manuscript, and keep the Table 2 as is. I don't consider this essential, but most journals refrain from showing the same data twice.

**Author Response: This section has been revised considering the Reviewer comments. Rather than convert the table to a figure, we have instead revised the text. The text now gives a summary of the findings and the important statistical result rather than restating all the data.**

348: inset space between "Figs 1" **Author Response: Accepted**

398: correct capitalization and punctuation. **Author Response: Corrected**

401 - 402 and 420 - 429: The following is optional for the authors to consider if they plan to submit a revised manuscript. I encouraged the authors to consider including bottle bioassays (BBA) for ReMoa Tri in hopes it would provide solid data that directly compares ReMoa Tri to insecticides that are used currently. While I appreciate the authors explanation for their absence is that there is currently no CDC or WHO BBA for ReMoa Tri, readers may find that explanation unsatisfying as BBA can be conducted with products that are applied in the field. While this isn't encouraged by CDC or WHO for current insecticide classes, ReMoa is a proprietary mix of active ingredients that are unlikely to be easily sourced separately any time soon for reconstitution in CDC-like BBA that utilizes technical grade ingredients (e.g., like permethrin synergized with PBO). In the absence of ReMoa Tri BBA, the authors should consider comparing the results of the ULV field application trials with ReMoa Tri to published ULV studies with permethrin, deltamethrin, malation, and/or naled. While mosquito populations in this and published studies are not identical, there are some that were done in areas with known insecticide resistance, and could be used to provide an approximate comparison of ReMoa Tri to other insecticides that are widely used. This recommendation is not to make preparing a revision unwieldy (and it is optional for authors to consider), but is aimed at helping the authors to provide a balanced analysis of ReMoa Tri vs current insecticide classes. ReMoa Tri seems to be a good product, but readers may be left wondering how it compares to the chemistries that are already on the market. A discussion of this could benefit the manuscript, but again, not required in my opinion.

**Author Response: We must reiterate that developing a BBA for this product was beyond the scope of the work that we undertook. First, the BBAs conducted on most of the collections in this study occurred before the ReMoa Tri formulation was available so it would have been quite impossible to test these populations with the product. Second, we did not have access to the formulation except for the field study under the specific terms of the EPA’s EUP.**

**As we noted in our previous response to reviewers about this issue at the first revision, we agree with this Reviewer that a bottle bioassay method is sorely needed but we exercise the option (given here by the Reviewer), not to include one in this study. It is an area for future work, and we indicate this in the manuscript (Lines 425-431).**

**We do also agree that we should have included a bit more context, so we have edited the text and cited a couple of recent field spray studies with Florida Culex populations to show that the IR seen in the lab is also observed in the field with commercial formulations. Even though these populations are not specifically those we tested, they do demonstrate that IR exists in many field populations to current formulations and give some baseline of how current formulations perform.**
